# *SH2D2A* Modulates T Cell Mediated Protection to a B Cell Derived Tumor in Transgenic Mice

**DOI:** 10.1371/journal.pone.0048239

**Published:** 2012-10-29

**Authors:** Tone Berge, Ingrid Helene Bø Grønningsæter, Kristina Berg Lorvik, Greger Abrahamsen, Stine Granum, Vibeke Sundvold-Gjerstad, Alexandre Corthay, Bjarne Bogen, Anne Spurkland

**Affiliations:** 1 Institute of Basic Medical Sciences, Department of Anatomy, University of Oslo, Oslo, Norway; 2 Centre for Immune Regulation, Department of Immunology, Oslo University Hospital Rikshospitalet and University of Oslo, Oslo, Norway; 3 Department of Neurology, Oslo University Hospital Ullevål, Oslo, Norway; MRC National Institute for Medical Research, United Kingdom

## Abstract

**Background:**

T cell specific adapter protein (TSAd), encoded by the *SH2D2A* gene, modulates signaling downstream of the T cell receptor (TCR). Young, unchallenged *SH2D2A*-deficient C57BL/6 mice exhibit a relatively normal immune phenotype. To address whether *SH2D2A* regulates physiologic immune responses, *SH2D2A*-deficient TCR-transgenic BALB/c mice were generated. The transgenic TCR recognizes a myeloma-derived idiotypic (Id) peptide in the context of the major histocompatibility complex (MHC) class II molecule I-E^d^, and confers T cell mediated resistance to transplanted multiple myeloma development *in vivo*.

**Principal Findings:**

The immune phenotype of *SH2D2A*-deficient C57BL/6 and BALB/c mice did not reveal major differences compared to the corresponding wild type mice. When challenged with myeloma cells, Id-specific TCR-transgenic BALB/c mice lacking *SH2D2A* displayed increased resistance towards tumor development. Tumor free TCR-transgenic *SH2D2A*-deficient mice had higher numbers of Id-specific single positive CD4+ thymocytes compared to TCR-transgenic wild-type mice.

**Conclusion:**

Our results suggest a modulatory role for *SH2D2A* in T cell mediated immune surveillance of cancer. However, it remains to be established whether its effect is T-cell intrinsic. Further studies are required to determine whether targeting *SH2D2A* function in T cells may be a potential adjuvant in cancer immunotherapy.

## Introduction

Recognition of antigens by T cells via the T cell receptor (TCR) leads to coordinated signaling pathways that ultimately activates the T lymphocytes. This recognition is crucial for efficient T cell mediated responses towards infectious agents as well as cancer. TCR signaling is initiated by lymphocyte-specific protein tyrosine kinase (Lck) mediated phosphorylation of immunoreceptor tyrosine-based activation motifs (ITAMs) on the CD3 chains of the TCR complex. The ζ-associated protein of 70 kDa (Zap-70) kinase is then recruited to the phosphorylated ITAMs leading to its activation. Subsequent signaling reactions are initiated by active Zap-70 and Lck, resulting in phosphorylation of several other proteins important for T cell activation, including adaptor molecules [1]. Adaptor proteins contain modular domains that allow them to mediate specific protein-protein and protein-lipid interactions, thus bringing effector molecules such as enzymes into close proximity to their targets [2].

T cell specific adapter protein (TSAd) is encoded by the *SH2D2A* gene and is expressed in activated T and NK cells, as well as in certain subtypes of endothelial and epithelial cells. TSAd harbors several protein interaction motives, including a Src-homology 2 (SH2) domain, a proline-rich region containing Src-homology 3 (SH3) ligands, and several tyrosine phosphorylation sites serving as SH2 ligands (reviewed in [3]). TSAd interacts via multiple binding sites with Lck and modulates its kinase activity [4]–[9]. Furthermore, upon stimulation with the CXCL12 chemokine, TSAd promotes phosphorylation of Itk, thereby affecting actin polymerization and migration of T cells [10].

Despite its presumed role in regulating Lck and Itk during T cell signaling, *SH2D2A*-deficient mice develop normally and have a normal distribution of double negative (DN), double positive (DP) and single positive (SP) thymocytes [11], [12]. However, when stimulated with soluble anti-CD3 or anti-CD3/CD28 antibodies, peripheral T cells from *SH2D2A*-deficient mice proliferate less vigorously compared to cells from control mice [11]. Both anti-CD3 and anti-CD3/CD28 stimulated *SH2D2A*-deficient T cells produce less of the Th1 cytokines IL-2 and IFN-γ than wild type T cells [11]–[13]. These cells also show decreased phosphorylation of signaling molecules (i.e. Zap-70, LAT and PLCγ1) [7], which could be due to poor activation of Lck in the absence of *SH2D2A*. With age, *SH2D2A*-deficient mice develop a lupus-like autoimmune disease, which has been suggested to be associated with defective T cell death *in vivo* [13].

**Figure 1:**
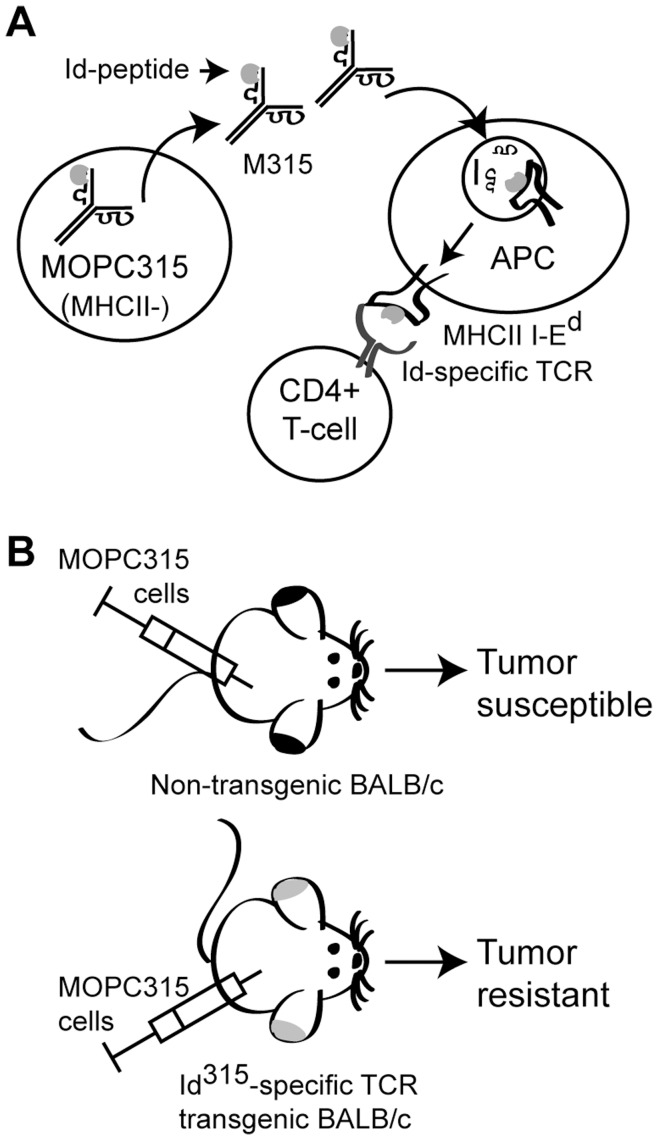
Schematic depiction of the MOPC315 model. (A) The MHCII negative MOPC315 cells secrete the IgA myeloma protein M315. This protein is endocytosed and processed by antigen-presenting cells (APCs), and CD4+ T cells with transgenic TCRs recognize a myeloma-derived idiotypic (Id^315^) peptide in the context of the MHC class II I-E^d^ molecule. (B) Normal BALB/c mice are susceptible to tumor development upon injection with MOPC315 cells, while mice with the Id^315^-TCR transgene are resistant towards tumor development. Resistance is mediated by tumor specific (Id-specific) TCR transgenic CD4+ T cells [16]–[19].

Although young, unchallenged *SH2D2A*-deficient mice do not display a strong immune phenotype, it has so far not been explored whether these mice display aberrant responses to immunological stimuli delivered in a physiological context. To investigate the role of TSAd in CD4+ T cell mediated cancer immunosurveillance, we crossed *SH2D2A*-deficient mice with idiotype (Id)-specific TCR-transgenic mice [14]. Such Id-specific TCR transgenic BALB/c mice are enriched for CD4+ T cells expressing TCRs recognizing a myeloma-derived Id peptide from the variable region of the MOPC315 myeloma secreted immunoglobulin light chain, presented on MHC class II I-E^d^ [14]–[16]. While non-transgenic mice develop fatal tumors, Id-specific TCR-transgenic mice are protected against development of experimental subcutaneous (s.c.) MOPC315 myelomas [17] (for schematic depiction of the model, see Figure 1). Protection is mediated by Id-specific CD4+ T cells, is independent of B and CD8+ T cells and requires secretion of the Id antigen [16]–[18]. Cancer eradication is achieved by an inflammatory reaction orchestrated by Id-specific Th1 cells [19]–[21].

The *SH2D2A*-deficient, Id-specific TCR-transgenic mice allowed us to study T cell responses triggered by the cognate peptide *in vitro*, as well as T cell mediated resistance to transplanted myeloma *in vivo*. Although the phenotypes of BALB/c mice as well as Id-specific TCR-transgenic BALB/c mice were not considerably affected by the lack of *SH2D2A*, the *SH2D2A*-deficient Id-specific TCR-transgenic mice displayed improved protection towards MOPC315 myeloma. This effect is at least partially dependent on T-cell specificity, since *SH2D2A*-deficient BALB/c mice with a normal T cell repertoire developed tumors to the same extent as wild-type BALB/c mice.

## Materials and Methods

### Ethical Statements

The animals were bred under conventional conditions, regularly screened for common pathogens and housed in compliance with guidelines set by the Experimental Animal Board under the Ministry of Agriculture of Norway. The research involving breeding of transgenic animals and experiments in transgenic and wild type animals was approved by the National Committee for Animal Experiments (Oslo, Norway).

### Mice and Cell Lines

*SH2D2A* deficient C57BL/6 and BALB/c mice were generated by backcrossing *SH2D2A* knockout mice on a C57BL/6–129 background (N8, kindly provided by Professor Jeffrey Bluestone [11]) to C57BL/6 and BALB/c mice (purchased from the Norwegian Institute of Public Health) for 2 and 10 generations, respectively, and then to homozygosity for the disrupted *SH2D2A* allele. Id-specific transgenic BALB/c mice were crossed with *SH2D2A*-deficient BALB/c mice. BALB/c mice heterozygous for both the *SH2D2A* null allele and the transgenic TCR were crossed with BALB/c mice heterozygous for the inactivated *SH2D2A* allele to generate littermates both for the studies of the TCR transgenic mice and the normal BALB/c mice with or without *SH2D2A* expression. A20 (obtained from American Type Culture Collection (ATCC)) and F9 (a BALB/c MHC class II positive A20/48B B cell lymphoma derived cell line that was transfected with Id [22]) were cultured in RPMI 1640 complete medium (RPMI 1640 medium supplemented with 10 % fetal calf serum (FCS), 1 mM HEPES, 1 mM non-essential amino acids, 1 mM sodium pyruvate, 1 mM L-glutamine, 100 units/ml penicillin, 100 µg/ml streptomycin (all from GIBCOBRL®, Life Technologies^™^) and 50 µM β-mercaptoethanol (Sigma)). MOPC315 cells [23] were cultured in complete RPMI 1640 medium without HEPES.

### Antibodies

Antibodies used were fluorescein isothiocyanate (FITC)- and phycoerythrin (PE)-conjugated rat anti-mouse CD4 (clone L3T4, Becton Dickinson (BD) Biosciences), SpectralRed (SPRD)-conjugated rat anti-mouse CD4 (clone L3T4, Southern Biotech), peridinin chlorophyll protein complex (PerCP)-Cy5.5-conjugated rat anti-mouse CD4 (clone RM4-5, BD Biosciences), FITC- and PE-conjugated rat anti-mouse CD8 (clone 53-6.7, BD Biosciences), FITC-conjugated CD44 (clone KM2011, Southern Biotech), PE-conjugated CD62L (clone MEL-14, Southern Biotech), PerCP-Cy5.5-conjugated hamster anti-mouse CD69 (clone HI.2F3, BD Biosciences), biotin-conjugated rat anti-mouse CD25 (BD Biosciences), PE-Cy7-conjugated rat anti-mouse CD25 (BD Biosciences), PE-conjugated rat anti-mouse CD45R/B220 (clone RA3-6B2, Southern Biotech), FITC-conjugated rat IgG1 (clone KLH-G1-2-2, Southern Biotech), PE-conjugated rat IgG2a (clone KLH G2a-1-1, Southern Biotech), PerCP-Cy5.5-conjugated hamster IgG (BD Biosciences), PE-Cy7-conjugated rat IgGλ1 (BD Biosciences) and PE- and biotin-conjugated transgenic TCR clonotype (GB113[24]). Secondary reagents used were streptavidin-CyChrom and streptavidin-Alexa647 (BD Biosciences). Furthermore, allophycocyanin (APC)-conjugated anti-TCRβ (H57–597, BD Biosciences), and PE-conjugated anti-CD5 (B19.1, Southern Biotech) were used in data not shown. Anti-FcγRII/III monoclonal antibody (mAb) (2.4G2, ATCC) was affinity purified in our laboratory.

### Analysis of Cells by Flow Cytometry

Single cell suspensions of lymph nodes, spleen and thymus were made by squeezing the organs through a cell strainer (70 µm nylon, BD Biosciences). Freshly isolated cells, or cells stimulated for indicated time points as described below, were stained as follows: unspecific binding was blocked by incubation with 100 µg/ml anti-FcγRII/III monoclonal antibody (mAb) prior to staining with specific mAbs. Biotinylated mAbs were detected with fluorochrom-conjugated streptavidin. Stained cells were analyzed on a FACSCalibur instrument with CELLQUEST (BD Biosciences) or FlowJo (Tree Star) software.

### Purification of CD4+ T Cells and in vitro CD4+ T Cell Stimulation

CD4+ T cells were isolated from single cell suspensions of spleen and lymph nodes by negative selection (Dynal® Mouse T Cell Negative Isolation Kit, Invitrogen). On average, the composition of the recovered population was more than 90 % CD4^+^ T cells (more than 96 % when isolated from lymph nodes) as analyzed by flow cytometry (FACSCalibur, BD Biosciences). Anti-CD3/CD28 stimulation: CD4+ T cells were stimulated with anti-CD3/CD28 beads (Dynabeads® Mouse T-Activator CD3/CD28 for cell expansion and activation, Invitrogen), bead: cell ratio = 1∶1 in complete RPMI 1640 medium. Alternatively culture plates were coated for 2 hours at 37°C with 1, 5 or 10 µg/ml anti-CD3 (clone 145.2c11), prior to cultivation with CD4+ cells in the presence of 1 µg/ml anti-CD28 (clone 37.51, BD Biosciences) in solution for 24 hours. Stimulation with antigen presenting responder cells: CD4+ T cells from TCR transgenic BALB/c mice were stimulated with irradiated (2000 rad) F9 or A20 cells. F9 strongly activates Id-specific CD4+ T cells [17], whereas the parental A20 cell line was used as a negative control. Ratio responder cells: stimulator cells = 1∶1 (80–200×10^3^ cells) in complete RPMI 1640 medium in 48 well plates.

### CFSE Proliferation Assay

To assess proliferation by flow cytometry, CD4+ T cells were labeled with 2.5–5 µM CFSE (Molecular Probes, Invitrogen) in PBS for 10 minutes at 37°C. Excess CFSE was removed by addition of fresh RPMI 1640 with 10 % FCS, followed by two additional washes with RPMI 1640 with 10 % FCS. CFSE labeled CD4+ T cells were stimulated as described above and CFSE dilution was analyzed by flow cytometry (FACSCalibur, BD Biosciences).

### *In vivo* Tumor Challenge

MOPC315 cells were cultivated to a cell density of approximately 0,1×10^6^ cells/ml, washed once in PBS prior to injection of 0,16×10^6^ (low dose) or 2×10^6^ (high dose) MOPC315 cells in 100 µl PBS s.c. into the right flank of mice (9–18 weeks old). Tumor development was monitored 2–3 times a week by palpation with a caliper in a blinded procedure, and volumes were calculated by the formula π/6×width^2^×length. A palpable tumor was defined to be at least 1 mm in width and length. To measure the level of the myeloma-secreted M315 protein in the serum, blood samples were taken once a week the first four weeks, then once every second week (in the experiments with 0,16×10^6^ MOPC315 cells) or on day 7 after tumor cell injection, and then every second week (in the experiment with 2×10^6^ MOPC315 cells). The M315 level was measured by ELISA as previously described [23]. In experiments with a low dose of MOPC315 cells: When the tumors exceeded 1 cm^3^, blood samples were collected, and the mice were sacrificed. At the end of the experiment (84 days after tumor cell inoculation), tumor-free surviving mice were sacrificed, and splenic CD4+ T cells were isolated and stimulated with F9 or A20 cells *in vitro* as described above. In experiments with a high dose of MOPC315 cells: When the tumors exceeded 1 cm^3^, blood samples were collected, and the mice were sacrificed. Cells from draining axillary lymph node, spleen and thymus were harvested to monitor the proportion of TCR transgenic (GB113+) CD4+(B220-) T cells in the different organs (thymocytes were also stained for CD8) as well as to monitor the surface expression of CD69 in the GB113+ CD4+ T cells from the draining lymph node.

### Statistical Methods

Statistical significant differences in population frequencies were assessed by two-tailed Mann-Whitney U test or paired or unpaired Student’s t-test. Differences in M315 myeloma protein concentrations were assessed by one-tailed Mann-Whitney U test. Differences in tumor resistance between groups of mice were assessed using two-tailed log rank test. P-values below 0.05 were considered significant.

## Results

### Small Changes in Thymocyte Proportions in *SH2D2A*-deficient Mice

**Figure 2:**
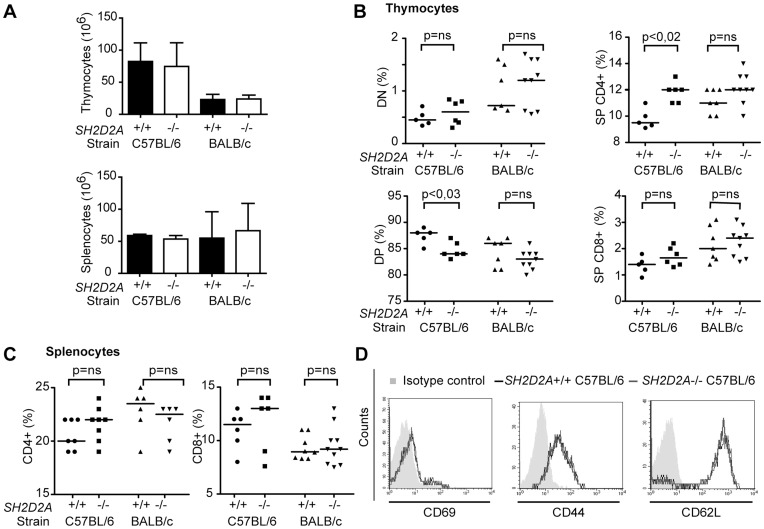
Small changes in thymocyte proportions in *SH2D2A*-deficient mice. (A) The graphs show the mean number of thymocytes (upper panel) and splenocytes (lower panel) with SD from *SH2D2A*-deficient C57BL/6 (7–16 weeks) and BALB/c (11–20 weeks) mice and corresponding age-matched controls. Student’s paired t-test showed no significant differences between the groups. (B, C) Thymocytes (B) and splenocytes (C) from C57BL/6 and BALB/c mice with indicated genotypes were labeled with anti-CD4 and -CD8 mAbs and their expression were monitored by flow cytometry. (B) The percentage of double negative (DN – upper left), double positive (DP – lower left), single positive (SP) CD4+ (upper right) and CD8+ (lower right) thymocytes is indicated for each genotype. (C) The percentage of CD4+ and CD8+ splenocytes is indicated in each group. (B, C) The median values are shown as lines in the diagrams P-values were calculated by the Mann-Whitney U test, p-values are indicated only when significant (p < 0.05). (D) Freshly isolated splenic CD4+ T cells were labeled with indicated mAbs prior to flow cytometry analysis. One representative experiment of at least four experiments performed on splenic CD4+ T cells from C57BL/6 mice (8–16 weeks old) is shown.

The *SH2D2A* null-allele was originally generated in 129 embryonic stem cells injected into C57BL/6 blastocysts [11]. Previous studies of *SH2D2A*-deficiency used mice backcrossed for 1–8 times against the C57BL/6 background [11]–[13]. Since genetic background may influence the phenotype, we backcrossed the *SH2D2A*-null allele [11] for 10 generations to the BALB/c as well as to the C57BL/6 strain. When the total number of thymocytes or splenocytes of *SH2D2A*-deficient mice (7–20 weeks old) were compared to that of age-matched wild type mice of the same genetic background, no significant differences were observed (Figure 2A). However, when examining the thymocyte proportions, a slight decrease in the percentage of DP thymocytes and a slight increase in the percentage of SP CD4+ thymocytes in *SH2D2A*-deficient C57BL/6 mice were found. The same trend was observed in thymocytes from *SH2D2A*-deficient BALB/c mice; however, the difference did not reach statistical significance (Figure 2B). Expression of the maturation markers CD5, CD69 and TCRβ were equivalent in normal and *SH2D2A*-deficient thymocytes irrespective of genetic background (data not shown). The proportions of peripheral CD4+ and CD8+ T cells in *SH2D2A*-deficient C57BL/6 mice was not significantly different from wild type mice, and the same was true for BALB/c mice (Figure 2C). In contrast to a previous report [13], we observed no alteration in the surface expression of CD69, CD62L and CD44 on *SH2D2A*-deficient peripheral CD4+ T cells neither in C56BL/6 (Figure 2D) nor BALB/c mice (data not shown). Taken together, our data indicate that *SH2D2A* may influence thymocyte selection. However, the effect is not large enough to result in alterations in the proportions of peripheral T cells.

### *SH2D2A-*deficiency does not Affect Anti-CD3/CD28 Induced CD4+ T Cell Proliferation or Expression of Activation Markers

**Figure 3:**
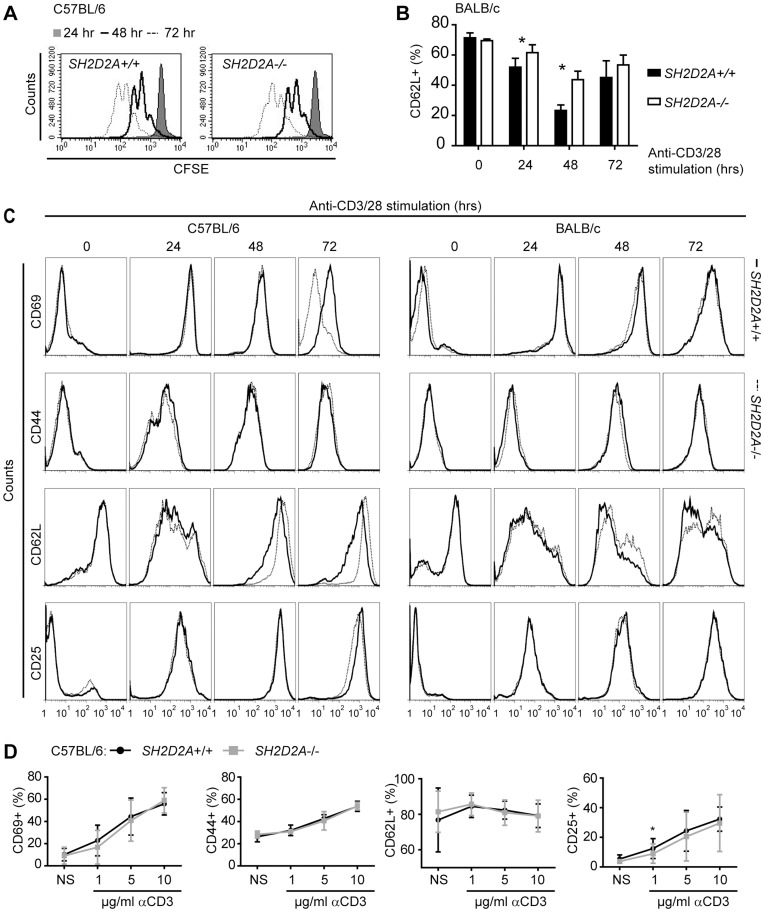
*SH2D2A*-deficiency does not have a major effect on anti-CD3/CD28 induced CD4+ T cell proliferation or expression of activation markers. (A) CFSE labeled CD4+ T cells from wild-type (*SH2D2A*+/+) and *SH2D2A*-deficient (*SH2D2A*-/-) C57BL/6 mice were cultivated with anti-CD3/CD28 beads as described in materials and methods. After 24, 48 and 72 hours, cells were harvested and the CFSE dilutions of *SH2D2A*+/+ CD4+ T cells (left diagram) and *SH2D2A*−/− CD4+ T cells (right diagram) were measured by flow cytometry. Grey area - CFSE profiles after 24 hours, open area (black line) - CFSE profiles after 48 hours and open area (stippled line) - CFSE profiles after 72 hours in culture with anti-CD3/CD28 beads. (B, C) Unlabeled CD4+ T cells from *SH2D2A*+/+ and *SH2D2A*−/− C57BL/6 (C, left side) and BALB/c (B and right side of C) mice were cultivated as in A. Prior to and after 24, 48 and 72 hours, cells were stained with CD69, CD44, CD62L or CD25 mAbs to assess T cell activation. (B) The percentage of CD62L positive CD4+ T cells from BALB/c mice is shown after indicated time points of anti-CD3/28 bead stimulation. P values were calculated with unpaired t-test, *p < 0.05. (C) FACS plots gated on CD4+ T cells from one representative experiment of at least three is shown. Solid line – *SH2D2A*+/+ CD4+ T cells, dashed line - *SH2D2A*−/− CD4+ T cells. (D) CD4+ T cells from *SH2D2A*+/+ and *SH2D2A*−/− C57BL/6 mice were cultivated with 1 µg/ml soluble αCD28 mAb and either 1, 5 or 10 µg/ml plate-bound αCD3 mAb for 24 hours prior to staining with the indicated mAbs. The percentage of positive cells is shown as a function of µg/ml of stimulating αCD3 mAb. NS – non-stimulated.

A previous report indicated that *SH2D2A*-deficient T cells proliferate less well upon stimulation with soluble antibodies against the TCR [11]. This conclusion was based on bulk analysis of tritium labeled cells that only quantifies overall cell division over a relatively narrow window of time. Analyses of CFSE-labeled CD4+ T cells by flow cytometry allow us to follow the division history of cell populations [25], [26]. Using this technique, we found that anti-CD3/CD28 beads induced strong and comparable proliferation of wild-type and *SH2D2A*-deficient CFSE-labeled CD4+ T cells after 48 hours of stimulation (Figure 3A and data not shown). Furthermore, activation-associated changes in surface expression of CD69, CD44, CD62L and CD25 were compared between *SH2D2A*-deficient mice and control mice of the same strain (Figure 3C). When combining the results from at least four experiments, we found no significant differences in the expression profiles of CD69, CD44 and CD25 between *SH2D2A* deficient and wild type CD4+ T cells from both mouse strains (data not shown). However, *SH2D2A*-deficient CD4+ T cells from BALB/c mice displayed a slightly smaller decline in CD62L surface expression after 24 and 48 hours in culture with anti-CD3/CD28 beads (Figure 3B and C), while this was not observed in C57BL/6 mice (data not shown). Of note, CD69 expression was routinely reduced in CD4+ T cells from *SH2D2A*-deficient C57BL/6 mice after 72 hours with αCD3/28 bead stimulation (Figure 3C, upper left panel), however, this difference did not reach statistical significance (Student’s paired t-test). When CD4+ T cells from C57BL/6 mice were stimulated for 24 hours with suboptimal doses of plate-bound αCD3 mAb in the presence of constant amount of soluble αCD28 mAb, *SH2D2A*-deficient CD4+ T cells displayed a significantly weaker CD25 response than wild type cells (Figure 3D). Taken together, *SH2D2A*-deficiency does not have a major impact on anti-CD3/CD28 induced CD4+ T cell responses *in vitro*.

### Characterization of *SH2D2A-*deficient Id-specific TCR-transgenic CD4+ T Cells

Stimulation of T cells with mAbs against CD3 and CD28 provides a rough estimate of the T cell responsiveness to mitogenic stimulation. However, *in vivo*, T cell activation is determined by a number of additional signals provided through co-receptors and adhesion molecules. To examine how *SH2D2A* might influence T cell responsiveness under more physiological conditions, we generated *SH2D2A* deficient BALB/c mice expressing a defined TCR. This transgenic TCR recognizes, in the context of MHC class II I-E^d^, an Id-peptide derived from the variable region of the immunoglobulin IgA that is secreted by the MHC class II-negative MOPC315 myeloma cells [15], [27] (Figure 1A).

**Figure 4:**
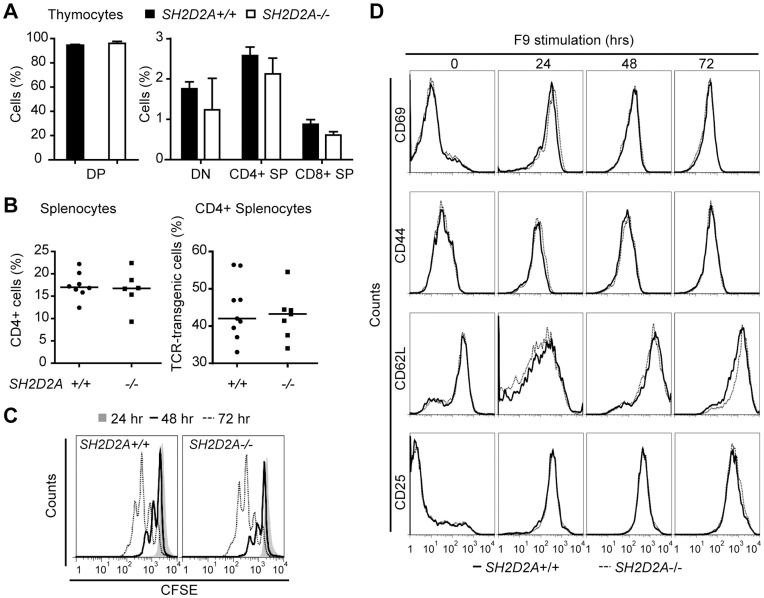
Characterization of *SH2D2A*-deficient Id-specific TCR-transgenic CD4+ T cells. (A) Thymocytes from Id-specific TCR-transgenic mice with indicated genotypes (9–15 weeks old) were labeled with anti-transgenic TCR (GB113), anti-CD4 and -CD8 mAbs. Expression of CD4 and CD8 on GB113+ thymocytes was monitored by flow cytometry. The mean average percentage with SD of double positive (DP), double negative (DN), single positive (SP) GB113+ CD4+ and CD8+ thymocytes is indicated for each genotype (n = 3). (B) Splenocytes from normal (*SH2D2A*+/+) and age-matched *SH2D2A-*deficient (*SH2D2A*−/−) mice (6–14 weeks old) were labeled with anti-CD4 and GB113 and expression was monitored by flow cytometry. Diagrams show the frequency of CD4+ T cells (left diagram) and the percentage of CD4+ T cells that express the Id-specific transgenic TCR (GB113+) (right diagram). The median values are shown as lines in the diagrams. (C) CFSE labeled CD4+ T cells from TCR transgenic *SH2D2A*+/+ (upper diagram) and *SH2D2A*−/− (lower diagram) were harvested after 24, 48 and 72 hours in culture with Id-positive F9, labeled with GB113 antibody. The CFSE dilution in GB113+ CD4+ T cells was measured by flow cytometry; grey area - CFSE profiles after 24 hours, open area (black line) - CFSE profiles after 48 hours and open area (stippled line) - CFSE profiles after 72 hours. One representative experiment out of four is shown. (D) Unlabeled CD4+ T cells from *SH2D2A*+/+ and *SH2D2A*−/− mice were cultivated as in C. Prior to and after 24, 48 and 72 hours, cells were stained with GB113 in addition to CD69, CD44, CD62L or CD25 mAbs to assess T cell activation by flow cytometry. FACS plots gated on GB113+ CD4+ T cells from one representative experiment of at least three are shown.

Absence of *SH2D2A* did not significantly alter the number of thymocytes or splenocytes in TCR transgenic mice (data not shown). Furthermore, the proportions of Id-specific thymocyte subsets or the Id-specific TCR-transgenic peripheral CD4+ T cells (Figure 4A and B) were unaffected. When CFSE labeled peripheral CD4+ T cells from TCR transgenic mice with or without *SH2D2A* were stimulated with Id-negative, MHC class II-positive (A20) antigen-presenting cells (APCs), no cell division or induction of cell surface activation markers was observed (data not shown). After 48 and 72 hours in co-culture with Id-positive, MHC class II-positive APC (F9 cells), the number of Id-specific CD4+ T cells that had undergone two and three rounds of cell division were similar irrespective of *SH2D2A* expression (Figure 4C). Upon co-culture with Id-positive APCs, *SH2D2A* deficiency did not affect the surface expression of the CD69, CD44, CD62L or CD25 activation markers on Id-specific CD4+ T cells (Figure 4D). Taken together, deficiency of *SH2D2A* does not appear to have an impact on activation and proliferation of TCR-transgenic CD4+ T cells in response to APCs expressing Id-I-E^d^ complexes.

### TCR Transgenic *SH2D2A-*deficient Mice are Resistant Towards Myeloma Development

**Figure 5:**
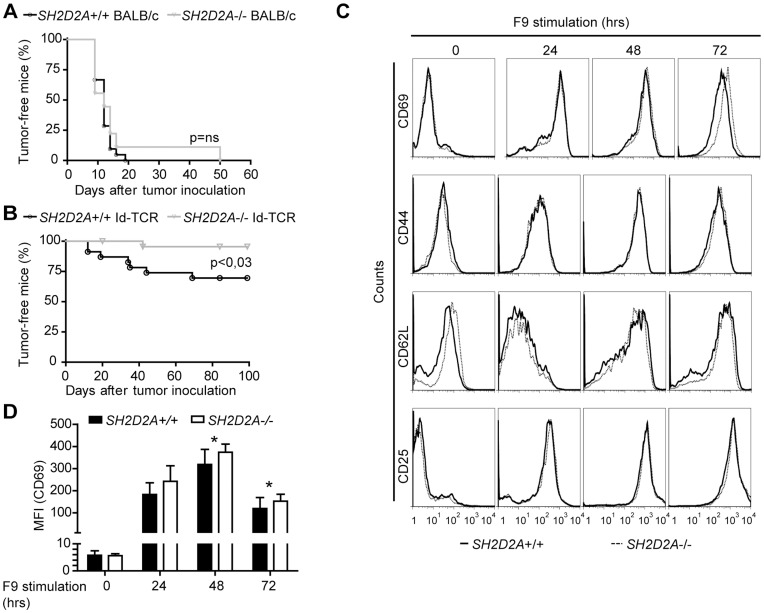
TCR transgenic *SH2D2A* −**/**− mice are resistant towards transplanted myeloma. Non-transgenic BALB/c and Id-specific TCR-transgenic (Id-TCR) BALB/c mice with (+/+) or without (−/−) *SH2D2A* expression were injected subcutaneously with low dose MOPC315 cells (160 000 cells). Tumor development was followed by palpation. (A, B) A tumor-free mouse was defined as a mouse that did not have a palpable tumor during the course of the experiment. The plots display tumor-take for (A) non-transgenic BALB/c mice with (n = 9) or without (n = 11) *SH2D2A* expression and (B) Id-specific TCR-transgenic BALB/c mice with (n = 23) or without (n = 23) *SH2D2A* expression. P values were calculated with two-tailed log rank test. Ns = non-significant.(C, D) Splenic CD4+ T cells were isolated from surviving tumor-free mice and stimulated *in vitro* with Id-positive F9 cells. Cells were labeled with GB113, recognizing the Id-specific transgenic TCR, and anti-CD69, CD44, CD62L or CD25 mAbs, prior to and after 24, 48 and 72 hours in culture with Id-positive cells. (C) FACS plots gated on GB113+ CD4+ T cells from one representative experiment are shown. (D) The diagram show the median value of the MFI (median fluorescent intensity) of CD69 at the indicated time points (n = 11) with SD. P-values were calculated with two-tailed, unpaired student t test, * indicate significant differences (at 48 hours: p = 0,003; at 72 hours: p = 0,03).

Having found that *SH2D2A*-deficiency was not associated with major changes in the *in vitro* responses of CD4+ T cells, we next set out to examine CD4+ T cell responses *in vivo*. As depicted in Figure 1, Id-specific TCR-transgenic mice are protected against MOPC315 tumors [17], and the primary anti-tumor immune response in these mice is mediated by the Id-specific TCR- transgenic CD4+ T cells [19]. Wild type and *SH2D2A*-deficient Id-specific TCR-transgenic mice as well as wild-type and *SH2D2A*-deficient non-TCR transgenic BALB/c mice were injected s.c. with a low dose of MOPC315 myeloma cells (0,16×10^6^ cells). Mice were examined for s.c. tumor growth by palpation. All non-transgenic BALB/c mice, independent of *SH2D2A*, developed s.c. myelomas (Figure 5A). As expected only 30 % (4 out of 14) wild type Id-specific TCR-transgenic mice got tumors. In contrast, none of the 13 *SH2D2A*-deficient Id-specific TCR-transgenic mice developed tumors. In a replicate experiment, 3 of 9 wild type Id-specific TCR-transgenic mice and 1 out of 11 *SH2D2A*-deficient Id-specific TCR-transgenic mice developed tumors. The overall result from the two experiments using low dose of MOPC315 cells showed that 30 % (7 out of 23) of the wild type Id-specific TCR-transgenic mice, and only 4% (1 out of 23) of the *SH2D2A* deficient Id-specific TCR-transgenic mice developed tumors (p<0.03, Figure 5B). Thus, *SH2D2A*-deficiency resulted in increased tumor resistance in Id-specific TCR-transgenic mice.

Since we did not observe any major effect of *SH2D2A* on the *in vitro* responses of naïve TCR-transgenic CD4+ T cells (Figure 4C and D), while there clearly was a difference in tumor protection (Figure 5B), we examined whether presence of *SH2D2A* affected the *in vitro* response of TCR-transgenic CD4+ T from tumor-experienced mice. CD4+ T cells were isolated from the spleen of surviving mice and stimulated with cognate antigen presented by APCs (F9 cells) *in vitro*. Expression of CD69 was moderately, but significantly, increased in *SH2D2A*-deficient TCR transgenic CD4+ T cells after two and three days of stimulation with F9 cells (Figure 5C and D), while no differences in the Id-response as assessed by proliferation (data not shown) or cell surface expression of CD25, CD44 and CD62L were observed (Figure 5C and data not shown). Conceivably, in this experiment with CD4+ T cells from tumor challenged mice, naïve Id-specific CD4+ T cells migrating from the thymus post tumor rejection could have contributed to the results, i.e. the observed responses may possibly have resulted from a combination of tumor-experienced T cells and recent thymic emigrants.

### TCR Transgenic *SH2D2A-*deficient Mice are also Resistant towards Tolerizing Amounts of Myeloma

**Figure 6:**
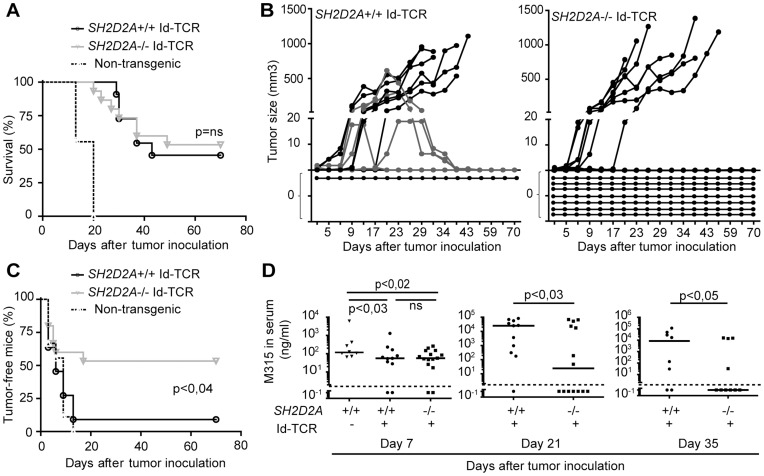
TCR transgenic *SH2D2A*-deficient mice are also resistant towards tolerizing amounts of myeloma. Id-specific TCR transgenic mice with (+/+, n = 11) or without (-/-, n = 15) *SH2D2A* expression were injected subcutaneously with high dose (2×10^6^) MOPC315 cells. Tumor development was followed by palpation (A–C) and M315 serum myeloma protein concentration was measured by ELISA 7, 21 and 35 days after tumor inoculation (D). (A) The plot display survival of Id-specific TCR-transgenic mice with and without *SH2D2A*. Mice with large tumors (tumor volume > 1 cm^3^) were euthanized. (B) The graphs display the tumor growth in the individual Id-specific TCR transgenic mice with (+/+, left diagram) and without (−/−, right diagram) *SH2D2A* expression. The grey lines highlight established tumors that were rejected. The horizontal lines at 0 mm^3^ visualize mice that did not develop a palpable tumor during the course of the experiment. (C) The plot displays tumor take of *SH2D2A*+/+ and *SH2D2A*−/− Id-specific TCR-transgenic mice. A tumor-free mouse was defined as a mouse that has not had a palpable tumor during the course of the experiment. (D) The plots present the M315 serum level 7, 21 and 35 days after tumor cell inoculation. Lines represent the median values of the M315 measurements. The dots below the dashed line (at 2 ng/µl) are below the detection limit of the ELISA assay. P values were calculated with two-tailed log rank test (A, C) and one-tailed Mann-Whitney U test (D). Ns = non-significant.

It has previously been observed that the tumor protective effect of Id-specific TCR transgenic CD4+ T cells can be partially overcome by injection of a greater number of myeloma cells, leading to tumor development in 60–80 % of wild-type TCR-transgenic mice [28]. We thus tested whether *SH2D2A*-deficient mice were still resistant towards myeloma when larger inoculums of myeloma cells were used. Normal non-transgenic BALB/c mice as well as wild type and *SH2D2A*-deficient Id-specific TCR-transgenic mice were injected subcutaneously with a high dose of MOPC315 myeloma cells (2×10^6^ cells). All wild type BALB/c mice, 55 % (6 out of 11) wild type Id-specific TCR-transgenic mice and 47 % (7 out of 15) *SH2D2A*-deficient Id-specific TCR-transgenic were sacrificed due to large tumor burden (>1 cm^3^) (Figure 6A). Additionally, four wild type Id-specific TCR-transgenic mice developed tumors that shrank and became impalpable prior to termination of the experiment (grey lines in Figure 6B). Thus, 53 % (8 out of 15) of the *SH2D2A*-deficient Id-specific TCR-transgenic mice and only 9 % (1 out of 11) of the wild type Id-specific TCR-transgenic mice remained free of clinically apparent tumors during the course of the experiment (p<0.04, Figure 6B and C). Since MOPC315 cells secrete M315 IgA, the amount of M315 in serum can be used as an indirect measurement for MOPC315-tumor load. M315 serum levels measured on day 21 and 35 after tumor cell injection, were significantly lower in *SH2D2A*-deficient compared to wild type Id-specific TCR-transgenic mice (Figure 6D). In summary, *SH2D2A*-deficiency led to increased tumor resistance of Id-specific TCR-transgenic mice also when they were challenged with tolerizing amounts of tumor cells.

### *SH2D2A-*deficient Mice have Increased Numbers of Id-specific TCR–transgenic SP CD4+ Thymocytes

**Figure 7:**
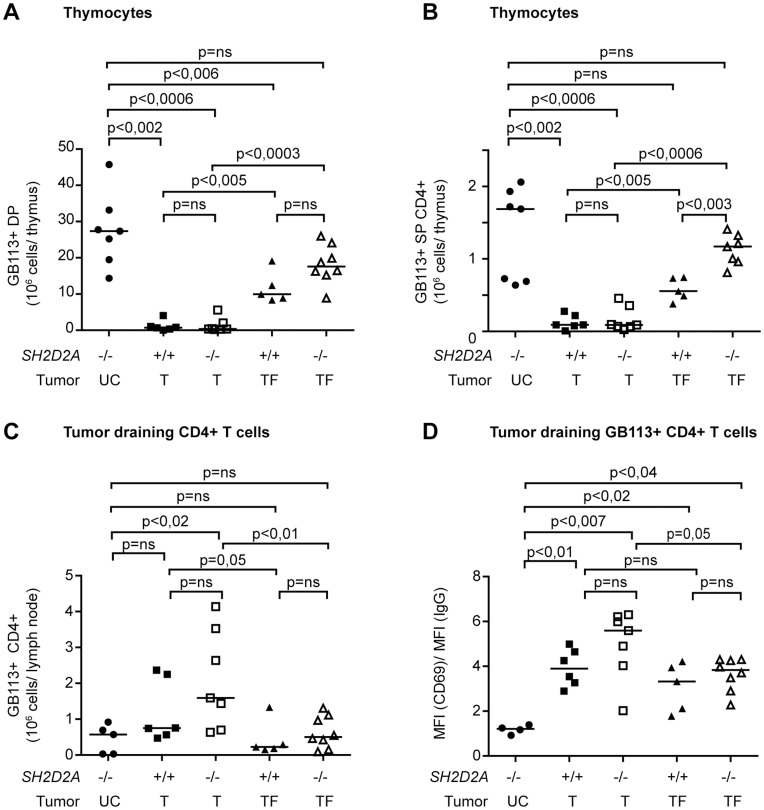
*SH2D2A*-deficient mice have increased numbers of Id-specific TCR–transgenic SP CD4+ thymocytes. The graphs represents from the left; “UC” –unchallenged mice, i.e. control Id-specific TCR-transgenic mice that did not receive tumor cells, wild-type (*SH2D2A*+/+) and *SH2D2A* deficient (*SH2D2A*−/−) mice that had to be sacrificed due to development of large tumors (tumor volume > 1 cm^3^) (T = tumor) or that was tumor free at the end of the experiment 70 days after tumor inoculation (TF = tumor free). These tumor-free mice never had a palpable tumor during the course of the experiment, or they had a tumor that never reached 1 cm^3^ and that disappeared prior to the end of the experiment. The median values are shown as lines in the diagrams. (A, B) Thymocytes from mice with indicated genotypes were labeled with anti Id-TCR (GB113), anti-CD4 and anti-CD8 mAbs and their expressions were monitored by flow cytometry. The total number of Id-specific TCR-transgenic (GB113+) double positive (DP, CD4+ CD8+) and single positive CD4+ (SP, CD4+ CD8−) thymocytes is indicated for each genotype. (C) The diagram display the number of GB113+ CD4+ T cells in the tumor-draining lymph node of the same mice as in A and B. Of note is that the number of GB113+ CD4+ T cells in unchallenged mice is very low due to the small size of the draining lymph node in the absence of tumor challenge. (D) The GB113+ CD4+ T cells from the draining lymph node were labeled with anti-CD69 mAb, and their expression was monitored by flow cytometry. The diagram shows the median fluorescence intensity (MFI) of CD69 divided by the MFI of isotype control in GB113+ CD4+ T cells. P values were calculated with two-tailed Mann-Whitney U test. Ns = non-significant.

In Id-specific TCR-transgenic mice, tolerance in the presence of the growing myeloma is associated with deletion of tumor-specific thymocytes and peripheral CD4+ T cells [28], [29]. Accordingly, TCR-transgenic mice with large tumors (T), had significantly reduced numbers of Id-specific (GB113 positive) DP (CD4+CD8+) and SP CD4+ thymocytes compared to tumor-free (TF) as well as unchallenged (UC) mice. In the presence of tumor, mice lacking *SH2D2A* showed a similar pattern of thymic tolerance induction as wild type mice (Figure 7A and B). However, tumor free *SH2D2A*-deficient mice displayed a significantly higher number of GB113 positive SP CD4+ thymocytes compared to tumor free wild-type mice (Figure 7B), indicating less extensive deletion of GB113 positive SP CD4+ thymocytes in the absence of *SH2D2A*. In the tumor-draining lymph node, *SH2D2A* did not affect the number of GB113+ CD4+ T cells (Figure 7C). However TCR transgenic *SH2D2A*-deficient peripheral CD4+ T cells in tumor mice displayed higher CD69 surface expression compared to mice without tumor (p = 0.05) or unchallenged mice (p<0,007) (Figure 7D).

Taken together, in the absence of *SH2D2A*, TCR-transgenic mice are better protected from transplanted myeloma, which correlates with reduced central deletion of tumor-specific SP CD4+ thymocytes in tumor-free mice.

## Discussion

The functional role of the *SH2D2A*-encoded adapter protein TSAd in T cells is not well understood. Despite its presumed role in regulating Lck and Itk during T cell signaling, unchallenged *SH2D2A*-deficient mice have no major immune defects. Here we report that *SH2D2A*-deficient TCR-transgenic mice display increased resistance towards transplanted myeloma.

TSAd modulates early TCR signaling events through its interaction with and modulation of the Src family kinase Lck [4], [5], [7], [8], [10]. *SH2D2A*-deficient mice display no gross alterations in immune phenotype, but reports on the details are conflicting [7], [11]–[13]. Some of these discrepancies may be explained by differences in genetic or environmental factors. The *SH2D2A* null gene was originally established on a 129 background [11]. To minimize the effect of mixed genotype, we backcrossed the *SH2D2A*-null gene onto C57BL/6 or BALB/c for 10 generations. Thymic development of T cells in these *SH2D2A* mice only showed minor alterations, and peripheral CD4+ T cells from these *SH2D2A*-deficient C57BL/6 or BALB/c mice responded similarly to CD4+ T cells from wild-type mice upon anti-CD3/CD28 stimulation. Moreover, we have not observed the age dependent autoimmune phenotype in these mice, as previously reported by Drappa et al [13] (Grønningsæter, manuscript in preparation). While backcrossing for 10 generations does not eliminate the possible presence of 129 derived alleles located close to the *SH2D2A* locus [30], our current data as well as previous reports strongly indicate that *SH2D2A* do not exert a major effect on the unchallenged immune system of the mice.

To explore the effect of *SH2D2A* deficiency in a more physiological context we used a well-characterized TCR-transgenic model for CD4+ T cell mediated resistance to the MOPC315 mouse myeloma [17], [19], [27]. The transgene encodes a TCR that recognizes an Id peptide from myeloma derived Ig in the context of I-E^d^. These Id-specific TCR transgenic mice are protected against transplanted myeloma, and the primary anti-tumor response is mediated by the Id-specific TCR transgenic CD4+ T cells [19]. The observation that Id-specific TCR-transgenic mice were more resistant to myeloma in the absence than in the presence of *SH2D2A* could be due to altered vascular function, as *SH2D2A* is also expressed in endothelial cells [31], [32]. In the T241 fibrosarcoma tumor model, *SH2D2A*-deficient C57BL/6 mice develop smaller tumors as a result of reduced angiogenesis [31]. However, since there was no difference in myeloma development between normal and *SH2D2A*-deficient BALB/c mice, the enhanced tumor protection observed in the *SH2D2A*-deficient Id-specific TCR-transgenic mice is difficult to ascribe to endothelial cells and angiogenesis alone. The possibility exists that the increased resistance of the *SH2D2A*-deficient Id-specific TCR-transgenic mice results from the combination of the expression in TCR-specific CD4+ T cells (with close to identical efficacy as those from *SH2D2A*-deficient mice) with the altered vascular function in *SH2D2A*-deficient endothelial cells [31]. However, this remains to be formally proven using chimeric animal models or conditional knock out mice.

The MOPC315 TCR-transgenic model is distinguished by the fact that the tumor antigen, M315 myeloma protein, is secreted by the tumor and is widely distributed in peripheral tissues including the thymus. Thus, we have previously found that upon exposure to a large tumor load, tumor development correlates with induction of both central [29] and peripheral [28] Id-specific tolerance. The reduced number of TCR-transgenic SP CD4+ thymocytes (Figure 7B) and reduced percentage of peripheral CD4+ T cells (data not shown) in mice with tumor compared to tumor-free mice, independent of *SH2D2A* expression, is consistent with these previous results. However, in the absence of *SH2D2A*, the numbers of GB113+ CD4+ SP thymocytes of tumor resistant mice were significantly higher than in the wild type mice (Figure 7B). This indicates that *SH2D2A* deficient cells are less susceptible to the tolerizing effect of a large tumor inoculum. However, using the current experimental set up, we can not exclude that *SH2D2A*-deficient mice very rapidly eliminate the MOPC315 tumors (before they are palpable), which would result in lower levels of M315 in their sera and consequently to less central tolerance.

We have recently shown that IFN-γ produced by Id-specific CD4+ T cells render macrophages directly cytotoxic to cancer cells [20] and thereby tumor protection. Although we did not measure IFN-γ production in this study, our observation that sub-optimal TCR stimulation elicited a lower response in *SH2D2A*-deficient CD4+ T cells, fits with previous reports that *SH2D2A*-deficient cells secrete less of the Th1 cytokines IL-2 and IFN-γ upon anti-CD3 stimulation [11]–[13]. It is thus less likely that the increased tumor protection observed in *SH2D2A*-deficient mice can be explained by more efficient IFN-γ production by the Id-specific CD4+ T cells.

The functional avidity of TCR controls positive and negative selection in the thymus [33]. There is a sharp threshold of TCR avidity in the thymus, whereby thymocytes become activated leading to activation induced cell death (AICD) [34]. Some reports indicate that *SH2D2A*-deficient T cells are less likely to go into AICD. While Drappa and colleagues reported that injection of superantigen did not lead to deletion of the targeted T cells in *SH2D2A*-deficient mice [13], this was not confirmed by another group [12]. In a classic model of negative selection in the thymus, *SH2D2A*-deficient H-Y TCR-transgenic male mice displayed increased numbers of both DP and SP CD4+ and CD8+ thymocytes compared normal mice [12], suggesting that negative selection in the absence of *SH2D2A* is not as efficient as in wild type mice. In the normal situation, clonal deletion in the thymus occurs late in development, at the DP-to-SP transition [35]. The small but significant increase in the proportion of SP CD4+ thymocytes in *SH2D2A*-deficient C57BL/6 mice is thus a further indication that T cells and thymocytes could be less susceptible to AICD in the absence of *SH2D2A*.

Activation of T cells is followed by clonal expansion and differentiation into recirculating central memory-like T cells and effector T cells. The latter subsequently undergo AICD coinciding with clearance of the antigen [36]. In the context of a growing tumor, T cells are chronically exposed to antigen. This may lead to T cell exhaustion [28], [37] and failure to control the tumor. Recently Caserta and colleagues showed that TCR-transgenic CD4+ T cells with reduced Lck levels were better able to control growth of a solid tumor, possibly because the reduced functional avidity of the T cells favored the persistence of cells with effector phenotype [38]. We and others have shown that TSAd modulate the activity of Lck [4], [5], [7]–[9]. The improved tumor resistance observed in *SH2D2A*-deficient Id-specific TCR-transgenic mice could thus be due to alteration in Lck activity mimicking reduced Lck levels, which results in persistence of effector T cells and improved tumor response as described by Caserta and colleagues [38]. We did not see an increase in effector CD4+ T cells in tumor free *SH2D2A*-deficient mice (CD44^high^, CD62L^low^, Figure 5C and data not shown). However, we observed a small but significant increase in CD69 expression in activated CD4+ T cells, when cells were stimulated *in vitro* after *in vivo* tumor challenge. This corresponds to the previous observation that older *SH2D2A*-deficient mice have high frequency of CD69 positive T cells [13], and suggests that in the absence of *SH2D2A,* T cells maintain a phenotype of recent activation for a longer period of time than normal.

To conclude, *SH2D2A*-deficient mice on two different genetic backgrounds only exhibited subtle alterations in T cell differentiation and activation. Nonetheless, *SH2D2A*-deficient Id-specific TCR-transgenic BALB/c mice displayed increased resistance towards a B cell derived tumor. The mechanism for this increased tumor resistance remains to be established.
